# AI for Clinical Competency Assessment: Scoping Review of Methods and Applications

**DOI:** 10.2196/92826

**Published:** 2026-08-14

**Authors:** Wenjia (Stella) Zhang, Benjamin B Daniels, Carol Mita, Hoang Nguyen, David B Duong

**Affiliations:** 1 Department of Global Health and Population, T. H. Chan School of Public Health, Harvard University, 677 Huntington Ave, Boston, MA, 02115, United States, 1 4254991079; 2 Program in Global Primary Health Care, Harvard Medical School, Harvard University, Boston, MA, United States; 3 Francis A. Countway Library of Medicine, Harvard Medical School, Harvard University, Boston, MA, United States; 4 Beth Israel Deaconess Medical Center (BIDMC), Partnership for Health Advancement in Vietnam (HAIVN), Hanoi, Vietnam; 5 Division of Global Health Equity, Brigham and Women's Hospital, Boston, MA, United States

**Keywords:** artificial intelligence, large language models, clinical competency assessment, health care quality, medical education, AI, LLM

## Abstract

**Background:**

Strengthening the global health workforce is central to achieving universal health coverage, but health systems cannot improve what they cannot measure. Valid and scalable assessment of clinical competency is essential for monitoring workforce readiness and ensuring that expanded service coverage translates into high-quality care. Traditional standardized patients, however, remain resource-intensive, difficult to scale, and vulnerable to evaluator-related bias. Recent advances in AI have enabled AI-led simulated standardized patients (SSPs) that may address these limitations.

**Objective:**

Although existing reviews have examined AI-SSPs broadly within medical education, their use as clinical competency assessment systems remains less well characterized. This scoping review aimed to address this gap by systematically mapping the scope, design features, and validation approaches of AI-SSP tools for clinical competency assessment.

**Methods:**

We registered the protocol prospectively with the Open Science Framework and conducted a scoping review following the Joanna Briggs Institute Manual for Evidence Synthesis. We searched MEDLINE, Embase, CINAHL Complete, Education Source, Web of Science Core Collection, Inspec, ERIC, and Cochrane CENTRAL, supplemented by manual searching. Eligible primary studies involved an AI-based SSP interaction that yielded a quantitative score of respondents' clinical competency. Two reviewers independently screened records and resolved conflicts through discussion. Data were charted on study characteristics and populations, frontend platform and interface features, backend AI architectures, performance scoring mechanisms, and tool evaluation methods and outcomes.

**Results:**

Of 5987 database records and 4 identified through other methods, 21 studies describing 20 unique AI-SSP systems were included. Studies were published between 2008 and 2026, with 15 (71%) published in 2024 or later. Most studies were conducted in high-income settings and involved medical students. Systems shifted over time from rule-based virtual patient to large language model–enabled conversational platforms after 2022. History-taking was the most frequently assessed competency, and checklist coverage was the most common scoring approach. Among studies comparing AI-generated scores with human ratings, findings were mixed: some reported moderate to strong agreement, while others showed score inflation and inconsistent performance. Most studies focused on feasibility; fewer evaluated large-scale use or sustained real-world implementation.

**Conclusions:**

This review is innovative in shifting attention from AI-SSPs as educational simulations to AI-SSPs as emerging infrastructures for clinical competency assessment. Unlike prior reviews of virtual patients or AI in medical education, this review maps not only system design and learner interaction but also which competencies are assessed, how scores are generated, and how the assessment evidence has been validated. AI-SSPs could make clinical competency assessment more scalable and potentially more standardized, although more evidence is still needed to determine whether they achieve validity and reliability across diverse learner or practitioner groups.

## Introduction

Universal health coverage (UHC) cannot be realized without adequate human resources for health—a recognition that has shaped the UHC agenda since its inception and integration into the Sustainable Development Goals [1]. Achieving UHC depends not only on expanding service coverage but also on ensuring that care is delivered safely, effectively, and consistently. Despite decades of investment, with an average of more than 70% of health system expenditures spent on human resources, the global health workforce continues to face persistent shortages and wide heterogeneity in skills and practice quality, especially in resource-constrained settings [2-4]. These workforce quality gaps have direct and severe consequences, contributing to preventable deaths and compromised patient trust in health systems. Globally, more than 3 million deaths occur annually due to unsafe care, with estimates suggesting that in low- and middle-income countries, as many as 4 in 100 people die from unsafe care [5]. Tackling health workforce challenges, as well as improving the efficiency of health systems worldwide, requires valid, timely, and scalable measures of clinical competencies.

Clinical competency assessments are commonly used in 2 main contexts: medical education, where they support learning and progression across undergraduate, graduate, and continuing medical education; and health system monitoring, where they help track the skills of practicing providers. Written knowledge tests, such as multiple-choice questions, offer one scalable and standardized approach to assessment. However, because they are inherently closed-ended, they often fail to capture the complexity and interpersonal dimensions of real clinical encounters [6]. To address this, performance-based assessments exist to offer a more authentic approach that evaluates skills more comprehensively, including history-taking, counseling, clinical reasoning, and professionalism. The “gold standard” performance-based assessment is the Objective Structured Clinical Examination (OSCE), in which learners interact with actors who role-play as standardized patients (SPs) and are scored using checklists or global rating scales by an observer on the side [7]. Yet, OSCE programs are widely recognized as resource-intensive, requiring SP recruitment and training, examiner time, facilities, and coordination, all creating barriers to frequent deployment, especially at scale or in low- and middle-income countries [8,9]. Large-scale health workforce assessments, such as the World Bank’s decade-long Service Delivery Indicators surveys, further illustrate both the value and difficulty of generating representative measures of provider readiness using clinical vignettes [9]. These efforts can cost millions of dollars and require complex field operations, which are especially difficult in the reduced funding environment for global health when governments and international organizations face more limited capacity for financing.

Besides their resource intensity, another major limitation of OSCEs is that the standardization of cases does not fully eliminate subjectivity in evaluation. Even when clinical scenarios, SP scripts, checklists, and rating forms are designed to create comparable testing conditions, learner scores may still be shaped by evaluator-related bias and other sources of variation. Recent literature has emphasized that fairness in medical education assessment requires greater attention to the sociocultural processes through which judgments are produced [10]. SP clinical scenarios may encode gendered or stereotyped assumptions, while the observation and the checklist completion components may be affected by the social identities of both learners and assessors [11,12]. Evaluator effects are also well documented, including halo effects, contrast effects, and systematic differences in stringency or leniency across examiners, with examiner stringency alone accounting for up to 29% of score variation in some OSCE settings [13,14]. Thus, the need for scalable assessment is inseparable from the need for fairer and more consistent assessment systems.

Recent advances in AI create an opportunity to develop conversational, case-based simulations that mimic human SP encounters, potentially improving scalability and reducing the risk of evaluator bias. AI, including large language models (LLMs), has the potential to engage learners or practitioners in naturalistic clinical encounters at scale, responding dynamically to history questions, laboratory test requests, and more based on prespecified case scenarios. Several reviews have examined adjacent areas, but none fully address the assessment-focused scope of this review. Earlier reviews of virtual patients in health professions education established that digital patient simulations can support knowledge acquisition and skills development, but these reviews largely evaluated virtual patients as educational interventions rather than as systems for competency assessment [15,16]. More recent reviews have focused on LLM-based virtual patients, describing their growing use for medical training [17-20]. Broader reviews of AI and generative AI in medical education have also summarized applications across curriculum design, feedback, and educational support while highlighting ongoing concerns about validity, implementation, and evidence quality [21-23]. However, these reviews do not distinguish whether the AI system functions as the patient, the assessor, the feedback generator, or all three; nor do they systematically characterize what clinical competencies are assessed, how learner performance is scored, whether scoring is human-generated or AI-generated, and what evidence supports reliability, validity, or implementation readiness. This gap is important because AI–standardized simulated patStandardized Simulated Patients (SSPs) are not only educational simulations but also may become infrastructures for more scalable, routine, and fairer competency assessment. A dedicated scoping review is therefore needed to map the designs, assessed domains, and scoring and feedback approaches of AI-SSP systems for clinical competency assessment.

Given these gaps, this scoping review aimed to map the existing literature on AI-enabled SSP systems for clinical competency assessment. Specifically, we sought to characterize (1) the study characteristics, populations, and clinical contexts in which AI-SSPs have been evaluated; (2) the system architectures, model types, platforms, and interaction designs used to simulate SP encounters; (3) the clinical competencies assessed; (4) the quantitative scoring and qualitative feedback approaches used, including whether scores were generated by humans, AI, or hybrid methods; and (5) the maturity of available evidence, including feasibility, usability, scoring accuracy, learning outcomes, and implementation evidence. By synthesizing these dimensions, this review identifies how AI-SSPs are currently being used for competency assessment and what further validation, design, and implementation research are needed.

## Methods

### Protocol and Registration

This scoping review was conducted according to the Joanna Briggs Institute Manual for Evidence with special attention to Chapter 10, Scoping Reviews [24,25]. The reporting of this review is aligned with the PRISMA-ScR (Preferred Reporting Items for Systematic Reviews and Meta-Analyses Extension for Scoping Reviews) checklist [26] and the PRISMA-S (Preferred Reporting Items for Systematic Reviews and Meta-Analyses Literature Search Extension) checklist (Checklist 1) [27]. Our protocol was registered prospectively with the Open Science Framework on June 10, 2025 [28].

### Eligibility Criteria

Studies were eligible for inclusion if they described an AI-based SSP system with a clinical competency assessment component. For this review, “assessment” was defined as the production of a quantitative score that could support comparison either within a learner over time or across learners. The score did not need to be AI-generated; studies were eligible if the simulation itself was AI-based and the resulting performance was scored by any method, including by AI or by human raters. Eligible systems also had to be implemented or evaluated as a functioning tool, prototype, or product. Purely conceptual frameworks, design proposals, and systematic reviews were excluded.

Inclusion criteria were as follows:

- Primary research source: Reported original empirical data.
- AI-based SSP: The interaction between the user and the SSP must be AI-based.
- Health professions context: Focused on learners or practitioners in medicine, nursing, pharmacy, or other health professions.
- Clinical competency focus: Involves clinical skills such as history-taking, clinical reasoning, diagnosis, counseling, and so on.
- Assessment component: Produced a quantitative competency score, either as part of the AI-SSP platform itself or assigned post hoc by human raters based on the AI-SSP encounter. Platforms used for formative assessments (“assessment for learning”) and summative assessments (“assessment of learning”) are both eligible.
- Any scoring source: The score could be generated by AI or human raters reviewing AI-simulated encounters.
- Functioning system: Described a functioning tool, prototype, platform, or product.

Exclusion criteria were as follows:

- Nonprimary source: Systematic review, editorial, commentary, opinion piece, or protocol without results.
- No AI-based SSP interaction: The SSP encounter was not AI enabled, for example, video-based or enacted by human SPs.
- No competency score: Unstructured, narrative feedback only, which does not allow for comparability across learners.
- No clinical competency focus: Did not assess clinical or health professional competencies.
- No functioning system: Described only a conceptual model, design proposal, or system architecture without evaluating a functioning tool or prototype.

### Information Sources

A comprehensive literature search was conducted by searching MEDLINE (National Library of Medicine, OVID), Embase (Elsevier, embase.com), Cumulative Index to Nursing and Allied Health Literature (CINAHL Complete, EBSCOhost), Education Source (EBSCOhost), and Web of Science Core Collection (Clarivate), covering the period from their inception to an initial search date of June 12, 2025. This search was updated on May 7, 2026, to expand the search vocabulary, with the addition of Inspec (Institution of Engineering and Technology, EBSCOhost), a technology-oriented database to identify articles from the engineering literature, and a second education database, the Education Resources Information Center (ERIC, EBSCOhost), as well as the Cochrane Central Register of Controlled Trials (Cochrane Library, Wiley) to identify unpublished trials. Each database was searched individually on the specified platform.

The databases searched included meeting abstracts and proceedings, dissertations, and reports, in addition to articles published in the scholarly literature. A supplementary manual search was conducted by reviewing reference lists of relevant review articles retrieved by the database searches and by conducting targeted searches in Google Scholar using keywords. No online or print resources were purposefully browsed, and no additional studies or data were sought from authors or experts.

### Search

The search strategies were designed and carried out by a health sciences librarian (CM) based on discussion with the research team and predetermined eligibility criteria. No substantial portions of strategies from previously published reviews were used. The search terms encompassed three concepts: (1) patient simulations of clinical case–based vignettes, (2) AI and LLMs, and (3) clinical competency assessment. Controlled subject vocabulary (ie, MeSH, Emtree, and subject headings from CINAHL, Education Source, ERIC, and Inspec) was included when available and appropriate. No language, search filters, or publication date limits were applied. The searches did not undergo peer review. Search reporting was guided by PRISMA-S recommendations. The exact search strategies from each database are provided in Multimedia Appendix 1.

### Selection of Sources of Evidence

Records from all database searches were imported into Covidence systematic review software (Veritas Health Innovation Ltd), where duplicates were removed prior to screening. Using Covidence, 2 reviewers (BD and WZ) independently screened titles and abstracts from the search results. Conflicts were resolved through discussion. Full texts were then retrieved and reviewed by the same 2 reviewers independently. Any discrepancies between reviewers were discussed among reviewers and resolved by discussion. A formal critical appraisal of the included studies was not conducted, as the goal was to map the extent of the literature.

### Data Charting

A structured data charting template was developed in Covidence to extract information from the included studies. The template captured study characteristics, study population, SSP system features, clinical scenario design, learner interaction modalities, feedback and scoring mechanisms, backend model architecture, and product evaluation methods and results. The charting form was developed iteratively and pilot tested on an initial set of included studies by the 2 independent reviewers (BD and WZ).

### Data Items

Extracted variables included general study information (study ID, title, authors, country, and relevant context on why the tool was developed); study population (target learner or practitioner group, health profession, training level, total number of respondents, and any reported demographic or clinical experience information); SSP system characteristics (tool name, frontend interface, online or offline support, and compatible devices); clinical scenario design (number and type of clinical scenarios and how they were developed or validated); scenario display features (whether the SSP was displayed using text, avatar, images, or other modalities); user interaction (respondent mode of input and available clinical actions); quantitative scoring (the type of scores used and the logic by which they were derived); qualitative feedback (the type of qualitative feedback and the logic by which they were generated); backend AI model (response generation model of SSP interaction, any prompting strategy for LLM-based systems, knowledge graphs for rule-based systems, and so on); language localization; and product evaluation (methods and results used to evaluate scoring accuracy and reliability and respondent experience and perceptions). Any unclear or missing information was clearly indicated as “not reported” with potential reasons specified.

### Synthesis of Results

Extracted data were first reviewed and synthesized through discussions between the 2 reviewers to reach consensus. Thematic analysis was then conducted using the completed data charting spreadsheet. Each extracted variable was tabulated and compared across studies, with themes developed inductively from observed patterns and summarized using frequency counts and narrative synthesis where appropriate.

## Results

### Study Selection

**Figure 1.**
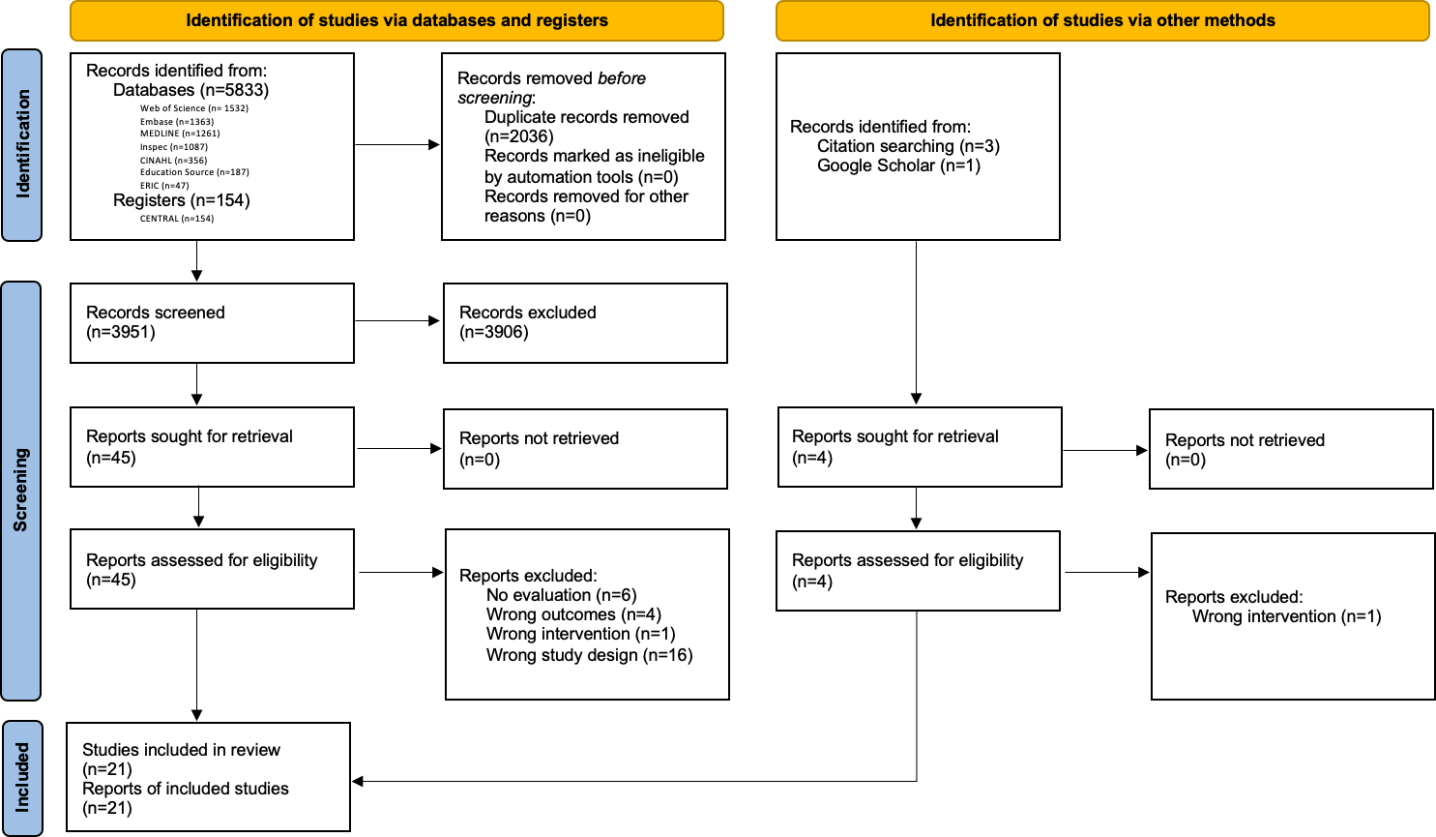
PRISMA (Preferred Reporting Items for Systematic Reviews and Meta-Analyses) flow diagram of studies selected.^29^ The diagram summarizes the number of records identified through database and supplementary searches, records removed before screening, records screened by title and abstract, full-text reports assessed for eligibility, studies excluded with reasons, and final studies included in the scoping review of AI-enabled simulated standardized patient for competency assessment in health professions.

A total of 5987 records were first identified through database searches and imported into Covidence. After removing 2036 (34%) duplicates, 3951 (66%) unique records were available for title and abstract screening. Two reviewers independently screened all titles and abstracts against the predetermined eligibility criteria, which focused on AI-based SSP systems with competency assessment components. Conflicts were resolved through discussion. At this stage, 3906 (98%) records were excluded because they did not meet the inclusion criteria. The full texts of 45 (1%) reports were then retrieved and assessed independently by the same 2 reviewers. Of these, 27 (60%) reports were excluded, with the most common reasons for exclusion including not involving an AI-based interaction with SP, no quantitative competency score produced, or not an empirical primary study. Four additional records were then identified through citation searching and Google Scholar, of which 3 (75%) studies were included. A final total of 21 studies were included in the final scoping review. The PRISMA flow diagram (Figure 1) provides further details on the screening process and reasons for exclusion.

### Study Characteristics

Table 1 summarizes the characteristics of the 21 studies included in this scoping review. The included studies were published between 2008 and 2026, with most studies published in recent years. Fifteen (71%) of the 21 studies were published in 2024 or later, reflecting the growth of AI-enabled SSP systems following the broader availability of LLMs. Earlier studies primarily evaluated rule-based or knowledge-based virtual patient systems, while all studies published after 2022 examined LLM-based SSPs.

**Table 1.** Study characteristics and populations.^a^

Study ID	Study title	Country	Study type	Study population	Sample size
Courteille et al, 2008 [30]	The use of a virtual patient case in an OSCE^b^-based exam - A pilot study	Sweden	Single-arm pilot feasibility evaluation	Fourth-year medical students in a 20-week clinical surgery course	N=110 participated in the ISP^c^ station (of 118 enrolled); 68 (62%) returned the ISP questionnaire; 47 video observed (p.e70-e72)
Oliven et al, 2011 [31]	Implementation of a web-based interactive virtual patient case simulation as a training and assessment tool for medical students	Israel	Prospective randomized crossover noninferiority study	Medical students at the start of clinical year in an introductory clinical course	N=262 across 3 y: 2008, n=87; 2009, n=76; and 2010, n=99. All took both the conventional human OSCE and the VP-OSCE^d^ in a randomized crossover design (p.236, Table 1).
Bond et al, 2019 [32]	Virtual standardized patient simulation: case development and pilot application to high-value care	USA	Single-arm pilot feasibility evaluation	First-year internal medicine and medicine-pediatrics residents	N=14 first-year residents; single arm (no control); 14 learners×8 cases=112 transcripts analyzed.
Maicher et al, 2019 [33]	Using virtual standardized patients to accurately assess information gathering skills in medical students	USA	Single-arm observational study with scoring validation substudy	First-year medical students early in medical interviewing curriculum	N=102 first-year medical students (single arm, observational). Scoring accuracy substudy: 20 randomly selected dialogues rated by 3 human raters+1 computer rater (ChatScript) (p.1054; p.1055).
Yang et al, 2019 [34]	MCRDR^e^ knowledge-based 3D dialogue simulation in clinical training and assessment	Australia	Proof-of-concept technical evaluation with user survey	Medical students and medical tutors	N=25 total: 20 medical students+5 medical tutors (students split into baseline n=10/explore n=10 for the interactive-efficiency test) (p.17).
Lee et al, 2022 [35]	Development of a chatbot to train physiotherapy students in clinical questioning and reasoning	Singapore	Development or proof-of-concept study	Year 1 physiotherapy students before clinical placement	Not reported—development or proof-of-concept paper; experimental evaluation explicitly stated as “ongoing,” no participant N given (p.477 abstract; p.480 sec.VI).
Brugge et al, 2024 [36]	Large language models improve clinical decision making of medical students through patient simulation and structured feedback: a randomized controlled trial	Germany	Randomized controlled trial	Medical students without clinical rotations; mostly early-semester learners	N=21 final (feedback: n=10; and control: n=11). 29 agreed; 5 dropped pre-randomization; 3 excluded post-randomization (technical data-save failures) (p.5).
Holderried et al, 2024 - e53961 [37]	A generative pretrained transformer (GPT)-powered chatbot as a simulated patient to practice history-taking: prospective, mixed methods study	Germany	Prospective mixed-methods single-arm feasibility study	Medical students and 2 midwifery students at a skills-refreshing event	N=28; single arm, no control. All 28 completed chatbot sessions and the Chatbot Usability Questionnaire (p.7).
Holderried et al, 2024 - e59213 [38]	A language model-powered simulated patient with automated feedback for history-taking: prospective study	Germany	Prospective validation study of automated feedback	Medical students, mostly third-year, during skills training course break	N=106 participated and completed≥1 conversation. 106 conversations, 1894 analyzable Q-A pairs after excluding 26 (p.5‐6).
Li et al, 2024 [39]	Leveraging large language model as simulated patients for clinical education	China	Technical system evaluation or human-LLM^f^ scoring validation	System or framework evaluators: nonmedical evaluators, clinical medicine students, senior physicians; no learner trial	SP quality eval: number of expert evaluators not reported (engaged 6 LLMs×8 cases, up to 20 rounds). Assessment validation: 80 dialogue records or 8 cases scored by 1 human expert. LLM-as-VD^g^: 9 human evaluators (3+3+3)×5 repeats×8 cases (p.3 sec.2.2; p.5‐6 sec.2.3‐2.4).
Chiu et al, 2025 [40]	Exploration of the role of ChatGPT in teaching communication skills for medical students: a pilot study	USA (inferred from author affiliations)	Single-arm pilot pre-post study	Second-year medical students	N=12; single-arm pre-post design; no separate arms (p.1872 Activity; p.1875 Multimedia Appendix 1)
Gorcilov et al, 2025 [41]	Large language models deliver interactive learning cases accurately and with rapid appropriate feedback	Australia (primary author); USA (co-authors)	Conference abstract; small pilot validation	Two junior physicians and 1 medical student acting as case users or investigators	N=3 learners (2 junior physicians+1 medical student); 5 cardiology cases; 250 LLM responses total. Pilot or proof-of-concept (p.S497-S498 Results).
Haut et al, 2025—AI Standardized Patient arXiv [42]	AI standardized patient improves human conversations in advanced cancer care	USA	Randomized controlled trial	Health professional students and practitioners: medical, PA^h^, nursing, residents, clinicians	N=51 (SOPHIE^m^: n=26; and control: n=25). Debrief survey completers 22/26 SOPHIE, 21/25 control; UI/UX^i^ survey 23/26 SOPHIE; poststudy interview 46/51; conversation reviews rated 506/510 (p.4 sec.2.1, Table 1).
Haut 2025—USIHC dissertation [43]	USIHC: understand, simulate, and improve human compassion in serious illness communication	USA	Dissertation; system development and randomized controlled trial	Medical, nursing, and PA trainees plus practicing clinicians	N=51 (SOPHIE intervention: n=26; control: n=25). 102 SP^k^ conversations recorded; 506/510 conversation ratings collected (p.72 sec.5.2.2; p.80 sec.5.4.1).
Liu et al, 2025 [44]	Development and validation of a large language model-based system for medical history-taking training: prospective multicase study on evaluation stability, human-AI consistency, and transparency	China	Prospective feasibility study	Third-year medical students with theoretical history-taking instruction but limited SP experience	N=31, single arm (no randomization or learner comparison groups; all used the fully processed AMTES^l^ system).
Qian et al, 2025 [45]	Use of large language models for rapid quantitative feedback in case-based learning: a pilot study	USA; Australia (multisite)	Pilot expert-comparison validation study	Medical student investigators using neurological cases	N=4 student investigators (p.1169).
Zheng et al, 2025 [46]	LLM-as-a-fuzzy-judge: fine-tuning large language models as a clinical evaluation judge with fuzzy logic	USA	Technical model validation or computational evaluation	Medical students	2303 conversation logs ->~2303 individual student utterances as data points; annotated by 7 expert judges. Split: 1611 train/231 val/461 test (p.5 sec.3.2; p.7 sec.3.5).
Zhou et al, 2025 [47]	Use of artificial intelligence to identify student behaviors associated with improved diagnostic accuracy of pulmonary diseases	USA (inferred from author affiliations)	Conference abstract; exploratory observational transcript analysis	Second-year medical students in pulmonary curriculum	602 unique student encounter transcripts; student headcount not reported—only encounter count n=602 (p.1).
Chen, 2026 [48]	AI-enhanced virtual reality simulation for nursing students’ empathy: automated scoring and interrater reliability in a randomized controlled study	Taiwan	Randomized controlled mixed methods study	Nursing students in obstetric nursing education	80 nursing students (experimental group: n=40; control group: n=40) (p.1 Abstract; p.2 sec.2.3; p.4 sec.3.1)
Hicke et al, 2026 [49]	MedSimAI: simulation and formative feedback generation to enhance deliberate practice in medical education	USA	Multisite pilot or quasi-experimental mixed methods evaluation	Medical students across 3 US institutions	410 unique learners; 1024 completed encounters. Inst A: 275 students/620 cases; Inst B: 104 students/362 cases; Inst C: 31 students/42 cases. Surveys: Inst B baseline n=85, exit n=19. Validation corpus: 104 OSCE transcripts (Inst C) rescored (p.6‐7 Table 1, sec.5.1).
Reynolds et al, 2026 [50]	AI versus human consensus: correlation of rubric-based scoring in pharmacy counseling	USA	Conference abstract; AI-human scoring correlation validation	Pharmacy students in a pharmaceutics class	Not reported (number of students or transcripts not stated in the conference abstract).

a Studies varied widely in design, ranging from single-arm pilot and feasibility evaluations to randomized controlled trials, validation studies, and technical model evaluations. Sample sizes also varied substantially, from small expert- or student-based pilots to larger multisite or multiyear deployments.

b OSCE: objective structured clinical examination.

c ISP: interactive simulated patient.

d VP-OSCE: Virtual Patient–Objective Structured Clinical Examination.

e MCRDR: Multiple Classification Ripple Down Rules.

f LLM: large language model.

g VD: virtual doctor.

h PA: physician assistant.

i UI/UX: user interface/user experience.

j SOPHIE: Standardized Online Patient for Healthcare Interaction Education.

k SP: standardized patient.

l AMTES: Artificial Intelligence-Powered Medical History-Taking Training and Evaluation System.

The included studies were conducted across a range of geographic settings, with the largest number from the United States or involving US-based study sites or author affiliations [32,33,40-43,45-47,49,50]. Other countries represented included Germany [36-38], China [39,44], Sweden [30], Israel [31], Australia [34,41,45], Singapore [35], and Taiwan [48]. Overall, the evidence base was concentrated in high-income countries, suggesting that the generalizability of findings across diverse medical educational contexts remains uncertain.

Study designs varied substantially, but most reflected early-stage development, feasibility testing, or validation rather than large-scale implementation research. Most studies conducted single-arm pilot feasibility testing [30,32,33,37,38,40,41,44,45,47,50]; others were framed as proof-of-concept, technical, or computational evaluations [34,39,46], while a limited number of studies used randomized controlled trials, crossover, or quasi-experimental designs [31,36,42,43,48,49]. Only 2 (10%) studies provided evidence beyond initial development or short-term evaluation [31,49]. Overall, the evidence base remained concentrated in pilot, feasibility, proof-of-concept, and validation work, with fewer controlled evaluations and large-scale implementation studies.

### Study Populations

Medical students across all academic years were the most frequently cited population, including students at the start of clinical training [31], first-year students in medical interviewing curricula [33], early-semester students without clinical rotation experience [36], second-year students [40], third-year students in history-taking training but with limited SP experience [44], and fourth-year medical students in surgery [30]. Several studies extended beyond medical students to include residents, junior physicians, practicing clinicians, or mixed groups of health professional trainees and practitioners [32,41-43]. Fewer studies examined other health professions learners, including physiotherapy students [35], midwifery students [37,38], nursing students [48], pharmacy students [50], and physician assistant trainees [42,43]. One technical evaluation study did not involve a conventional learner trial and instead evaluated AI-SSP quality and scoring using a combination of nonmedical evaluators and senior physicians. Overall, the included studies primarily centered on medical education rather than clinicians in practice, with more limited but emerging representation from nonmedical health professions.

Sample sizes and units of analysis varied widely across studies. Among studies reporting learner-level participation, sample sizes ranged from very small pilot studies with 3 to 4 participants [41,45] to larger evaluations involving more than 100 learners [30,31,38,49], and in one case, 410 unique learners across 3 institutions [49]. Several studies reported encounter-, transcript-, utterance-, or dialogue-level data rather than only learner-level sample sizes, including 112 transcripts [32], 80 dialogue records [39], 602 student encounter transcripts [47], 1024 completed encounters [49], and more than 2300 conversation logs or students’ utterances [46]. Some studies did not clearly report learner sample size or were development focused without a completed learner evaluation [35,50].

### AI-SSP System Architecture and Interaction Design

#### Systems Overview and Intended Use

As summarized in Table 2, the included studies evaluated a diverse set of AI-enabled SSP systems, ranging from dialogue simulation platforms to immersive virtual reality environments. Several studies involved formally named platforms, such as USC Standard Patient [32], CureFun [39], SOPHIE [42,43], AMTES [44], Turing Interactive Case Learning [45], 2-Sigma [46,47], VR-AI-ECS [48], and MedSimAI [49], while others described the intervention more generally as a “virtual patient,” “simulated patient chatbot,” or “AI-supported online platform” [31,33,35-38,40,41,50]. Almost all studies justified their systems by pointing to the same underlying problem: traditional SPs are resource intensive, costly, logistically difficult, and hard to scale. The systems varied in format and implementation, but most were designed to create repeatable clinical encounters in which learners could practice, receive feedback, or be assessed in a standardized environment. Overall, 20 unique AI-SSP systems were identified across 19 unique study teams, with 1 system evaluated by 2 studies [42,43] and 1 study team evaluating 2 variations of a system [37,38].

**Table 2.** AI-enabled simulated standardized patient (AI-SSP) architecture and interaction design.^a^

Study ID	AI-SSP system name	Primary purpose	Access and device compatibility	System architecture	Clinical scenarios	Scenario development	SSP display features	Respondent mode of input	Available clinical actions
Courteille et al, 2008 [30]	No proprietary name reported, referred to as “ISP (Interactive Simulation of Patients)"; developed at VP-Lab, Karolinska Institutet (p.e67, p.e76)	Assessment	Offline; computer	Rule-based or NLP	1 colorectal cancer surgical case	Adapted from existing ISP case; redesigned or validated by surgeons and senior clinicians	Avatar; images	Typed free text+structured actions	History-taking; visual examination; laboratory tests; clinical examination; and other
Oliven et al, 2011 [31]	No proprietary name reported, referred to as “Web-Based Virtual Patient (VP) System/VP-OSCE” (p.233)	Education + assessment	Online; computer	Rule-based or NLP	25 complaint prototypes with multiple cases	Faculty-authored prototype cases; keyword lexicon expanded through use	Text or web case interface	Typed free text+structured actions	History-taking, visual examination, laboratory tests, and clinical examination
Bond et al, 2019 [32]	USC Standard Patient (virtual standardized patient platform; open-source, standardpatient.org; USC Institute for Creative Technologies, 2014‐2017) (p.242 Platform).	Education + assessment	Online; computer	Rule-based or NLP	8 high-value care cases across 4 complaints	Faculty or resident-authored templates; iterative NLP mapping refinement	Avatar; text	Typed free text	History-taking, visual examination, chart review, laboratory tests, clinical examination; other: differential diagnosis and treatment
Maicher et al, 2019 [33]	No proprietary name reported, referred to as “Virtual Standardized Patient (VSP) system” (p.1053 Abstract; p.1054).	Education	Online or web; and computer	Rule-based or NLP (ChatScript)	15 VSP cases; study analyzed 1 low-back-pain case	Faculty-defined history elements using Kalamazoo checklist	3D avatar	Typed free text	History-taking
Yang et al, 2019 [34]	No proprietary name reported, referred to as “MCRDR Knowledge-Based 3D Dialogue Simulation System (“3D dialogue simulation system”)” (p.1‐2).	Education + assessment	Offline; computer	Rule or knowledge-based	GI^b^ or respiratory cases; ≥14 conditions	Tutor-authored from clinical samples or databases; MCRDR^c^ rules	3D avatar; text	Typed free text	History-taking, visual examination, laboratory tests, and clinical examination
Lee et al, 2022 [35]	No proprietary name reported, referred to as “Chatbot Virtual Patient (VP)/speech-enabled AI chatbot” (p.477‐478)	Education	Online; computer or speech-enabled web platform	Rule-based or NLP (DialogFlow)	1 musculoskeletal elbow-pain case	Student-team script supervised by faculty; iteratively refined	Text; speech-enabled chatbot	Speech	History-taking
Brugge et al, 2024 [36]	No proprietary name reported, referred to as “chatbot”	Education	Online; computer	LLM (GPT-3.5)	4 neurological or neurosurgical emergency cases	Contextual prompts; limited case details in prompt	Text	Typed free text	History-taking; other: differential diagnosis
Holderried et al, 2024 [37]	No proprietary name reported, referred to as “chatbot”	Education	Online; computer	LLM (GPT-3.5)	1 T2DM^d^-like history-taking case	Expert-authored illness script loaded into prompt	Text	Typed free text	History-taking
Holderried et al, 2024 [38]	No proprietary name reported, referred to as “chatbot”	Education	Online; computer	LLM (GPT-4)	1 reused history-taking illness script	Expert-authored illness script; feedback categories derived from script	Text	Typed free text	History-taking
Li et al 2024 [39]	CureFun	Education + assessment	Online; computer	LLM+RAG or knowledge graph (multiple LLMs)	8 Chinese SP^e^ cases across GI, endocrine, and pulmonary conditions	Scripts from an industry dataset cleaned and converted into case graphs	Text	Typed free text	History-taking, laboratory tests, and clinical examination
Chiu et al, 2025 [40]	No custom-built application, used ChatGPT (version 3.5) via the public web interface (p.1871 Abstract; p.1872 Activity)	Education	Online; computer	LLM (ChatGPT-3.5)	1 breaking-bad-news diabetes scenario	Research team scenario; student-authored prompt; SPIKES framework	Text	Typed free text	Other: breaking bad news
Gorcilov et al, 2025 [41]	No proprietary name reported, referred to as “an online platform using OpenAI’s GPT-4o” (p.S497 Method).	Education	Online; computer	LLM (GPT-4o)	5 cardiology cases	Investigator-developed cases; details limited	Text	Typed free text	History-taking; laboratory tests; and clinical examination
Haut et al, 2025 [42]	SOPHIE (Standardized Online Patient for Healthcare Interaction Education)	Education	Online; computer	Hybrid LLM+classifier (GPT-3.5-turbo)	3 advanced-cancer SIC^f^ cases	Palliative or oncology expert-authored; adapted from validated SIP^g^/VOICE^h^ cases	Avatar; voice	Speech	Other: SIC, guide through different treatment decisions
Haut, 2025 [43]	SOPHIE (Standardized Online Patient for Healthcare Interaction Education)	Education	Online; computer	Hybrid LLM+classifier (GPT-3.5-turbo)	Stage IV cancer communication modules+3 RCT^i^ cases	URMC^j^ faculty and validated SIC frameworks; actors or SP training	Avatar; voice	Speech	Other: SIC
Liu et al, 2025 [44]	AMTES (Artificial Intelligence-Powered Medical History-Taking Training and Evaluation System)	Education + assessment	Online; electronic device	LLM (DeepSeek-V2.5; Qwen-Max tested)	3 history-taking cases: respiratory, urinary, and GI	Senior clinical experts; aligned to licensing syllabus; and iteratively refined	Text	Typed free text	History-taking
Qian et al, 2025 [45]	Turing interactive case learning (platform name in Figure 2, p.1170); powered by GPT-4o.	Assessment	Online; computer	LLM (GPT-4o)	5 neurology cases	Faculty or expert-scored cases; development details limited	Text	Typed free text	History-taking; laboratory tests, clinical examination, and other: preliminary diagnosis
Zheng et al, 2025 [46]	LLM-as-a-Fuzzy-Judge (underlying clinical simulation platform: “2-Sigma”) (p.1; p.4 sec.3.1)	Assessment	Online platform; device not specified	LLM or fine-tuned judge (multiple backbones)	Wide range of 2-Sigma clinical dialogue scenarios	2-Sigma platform scenarios; and details limited	Text	Typed free text	History-taking; laboratory tests; clinical examination; and other
Zhou et al, 2025 [47]	2-Sigma platform (AI-based; University of Cincinnati College of Medicine) (p.1)	Assessment	Online platform; device not specified	Generative AI or multiagent (model not reported)	3 pulmonary cases: OSA^k^, COPD^l^, and PE^m^	Predefined 2nd-year curriculum framework; and details limited	Text	Typed free text	History-taking; and clinical examination
Chen, 2026 [48]	Virtual Reality Integrated Artificial Intelligence Empathic Communication Simulation (VR-AI-ECS)	Education	VR^n^-based; device not fully specified	AI dialogue engine (model not reported)	Obstetric empathy scenarios across childbirth care	Expert-informed prompts; grounded in empathy and experiential-learning models	VR; voice; and role-switching	Speech+embodied VR interaction	Other: users alternate between the roles of a pregnant woman and a nurse to experience both perspectives of maternity care
Hicke et al, 2026 [49]	MedSimAI	Education + assessment	Online; computer or voice supported	LLM (GPT-4o; OpenAI Realtime API)	Instructor-configurable diverse cases	Multi-institution SME^o^ co-design; structured templates or checklists	Text; and voice option	Typed or speech input	History-taking
Reynolds et al, 2026 [50]	No proprietary name, referred to as “investigational board-approved automated AI grading agent”(p.S1809).	Assessment	Text-based chatbot; and device not specified	Specific AI or LLM; model not reported	Pharmacy counseling cases	Preauthored cases; development details not reported	Text	Typed free text	Other: drug counseling

a Across studies, systems varied from early rule-based or traditional natural language processing (NLP) virtual patient platforms to more recent large language model (LLM)–based, retrieval-augmented generation (RAG)–supported, hybrid LLM classifier, and virtual reality systems.

b GI: gastrointestinal.

c MCRDR: Multiple Classification Ripple Down Rules.

d T2DM: type 2 diabetes mellitus.

e SP: standardized patient.

f SIC: serious illness communication.

g SIP: Study of Individualized Patient Care.

h VOICE: Values and Options in Cancer Care.

i RCT: randomized controlled trial.

j URMC: University of Rochester Medical Center.

k OSA: obstructive sleep apnea.

l COPD: chronic obstructive pulmonary disease.

m PE: pulmonary embolism.

n VR: virtual reality.

o SME: subject matter expert.

Across these 20 unique AI-SSP systems, their primary intended use fell into 3 broad categories. The largest group, 9 (45%) of 20 systems, framed AI-SSPs primarily as deliberate practice or educational tools, emphasizing safe, repeatable, and low-stress environments for learners to practice history-taking, communication, empathy, counseling, or case-based clinical reasoning [33,35-38,40-43,48]. While they do not list assessment as a primary purpose, they were included in this review because they all produce some form of quantitative score, and their functionality demonstrates strong potential for direct adaptation to assessment purposes. Six (30%) of 20 systems were positioned as hybrid training and assessment tools, combining SP interaction with structured feedback and scoring [31,32,34,39,44,49]. The remaining 5 systems focused explicitly on assessment using AI-SSP systems to evaluate user performance. Taken together, the literature suggests that AI-SSPs, even the ones with scoring or assessment components, have most often been introduced as formative learning environments. However, there exists an increasing subset of systems that explicitly emphasize assessment purposes, including automated scoring, structured feedback, and performance analytics.

#### Backend Architecture and AI Model

**Figure 2.**
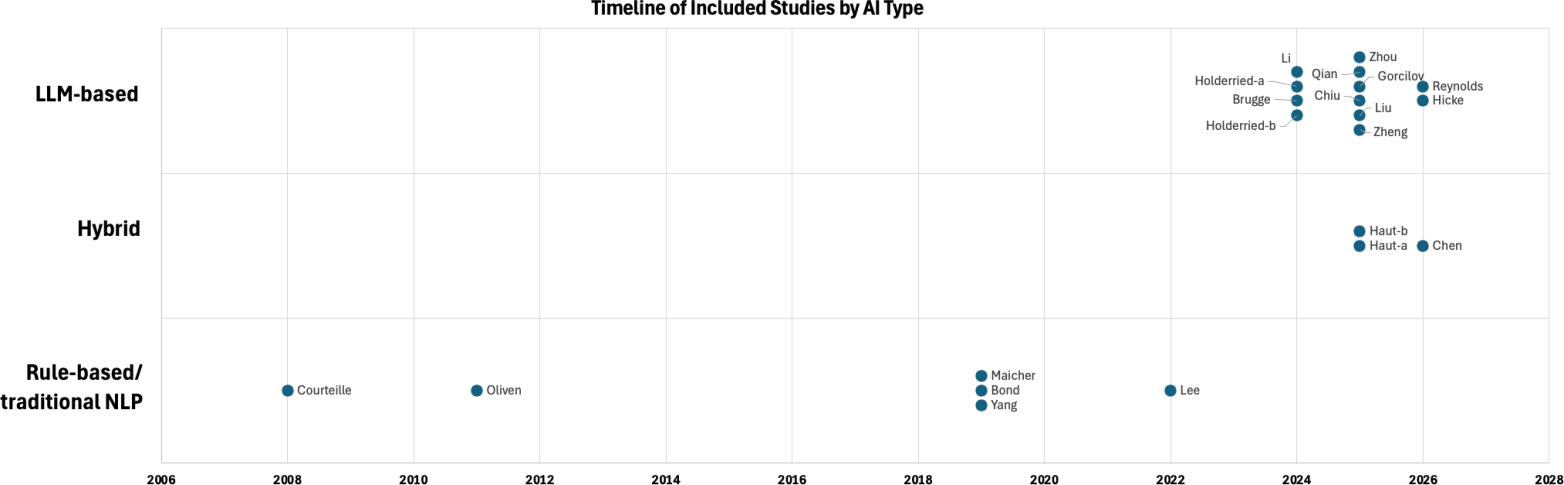
Timeline of included studies by AI model type. This figure maps the included studies by publication year and underlying AI model type, showing the field’s transition from earlier rule-based or traditional natural language processing (NLP) systems to more recent large language model (LLM)–based and hybrid AI approaches.

Backend architectures varied substantially across studies and showed a clear temporal shift in model type, as summarized in Figure 2. Earlier AI-SSP systems, particularly those published before 2022, relied on rule-based natural language processing, scripted dialogue structures, decision trees, keyword matching, or intent classification approaches. These systems used preauthored cases and expected learner inputs to be matched against predefined system response pathways, making the interaction more predictable but less conversationally fluid. For example, Maicher et al [33] used approximately 2500 ChatScript rules to support the virtual patient dialogue, Yang et al [34] developed a knowledge-based 3D dialogue system using classification rules, and Lee et al [35] used DialogFlow intents to classify learner questions and return scripted patient responses. These earlier architectures emphasized control, standardization, and reproducibility but were constrained by the range of prespecified utterances, intents, and response rules built into the system.

After 2022, all included studies either partially or fully relied on LLMs to power patient dialogue, scoring, or feedback generation. In contrast to rule-based systems, LLMs are AI models trained on large text datasets to interpret and generate natural language, which allows them to support more open-ended, contextually responsive SSP dialogue. Specifically, 2 (10%) studies used the public ChatGPT interface directly, representing a relatively low-barrier implementation model in which learners interacted with an existing chatbot rather than a fully custom AI-SSP platform [36,40]. In a study by Brugge et al, contextual prompts were entered into ChatGPT right before participants arrived at the computer, while Chiu et al instructed students to write their own prompt specifying that ChatGPT should act as a patient before they began the dialogue.

The majority of the newer studies, however, embedded LLMs within more controlled custom platforms or structured prompt pipelines rather than using an open-ended public chatbot interface. These systems typically combined a system prompt with case information, behavioral instructions, and specific constraints to define how the simulated patient should respond. For example, Holderried et al [37,38] used a 2-part prompt with behavioral instructions and illness scripts, Haut/SOPHIE [42,43] used serious illness communication scenarios with target skills such as agenda setting and responding to emotion, and Hicke et al/MedSimAi [49] used instructor-provided templates to build system prompts and separate evaluation prompts with JSON output. Several other studies also separated the simulated patient prompt from the scoring or feedback prompt, indicating that the same LLM was not simply prompted once to “be a patient” but was instead used through multiple task-specific prompt layers [37,38,40,42-44,49].

A smaller subset of studies incorporated additional backend structures to constrain LLM output, improve consistency, and reduce hallucinations. These included retrieval-augmented generation, structured case graphs, templates, or guardrails that limited the information available to the model and specified how the virtual patient should behave. For example, Li et al [39] used a structured case graph so that the model could retrieve relevant patient facts before generating a response, Haut/SOPHIE [42,43] aligned dialogue with target communication frameworks, and Hicke et al [49] used templates specifying patient demographics, history, communication style, emotional state, and vocabulary. These approaches suggest that newer AI-SSP systems are mostly “LLM wrapped” rather than fully open-ended. The model generates naturalistic conversation, but the architecture has constraints in place for what information it can use, how the patient should respond, and what learning or assessment goals the interaction is intended to support.

#### Clinical Case Scenarios and Their Development

The number of simulated cases ranged from 1 to 25 across studies. Most used a relatively small set of cases rather than a large clinical case bank [30,33,35,38,40]. The clinical content spanned multiple domains, including gastrointestinal and metabolic conditions, cardiopulmonary and respiratory presentations, neurology and neurosurgery, musculoskeletal complaints, oncology and cancer communication, obstetric and nursing empathy scenarios, and pharmacy counseling.

Earlier systems often used cases to assess biomedical clinical reasoning and diagnostics [30-32,34,36,45,47]. Later studies increasingly expanded case content to include communication-oriented and interpersonal competencies, including empathy, breaking bad news, counseling, professionalism, serious illness communication, and patient-centered communication behaviors [40,43,46,48-50]. Across studies, cases typically included patient demographics, chief complaint, symptom history, and expected learner tasks, with some systems also incorporating physical examination findings, investigation results, differential diagnosis, treatment decisions, emotional context, and grading rubrics.

Case development was mostly expert-led or faculty-led rather than AI-generated. Fourteen (67%) studies reported cases that were authored, adapted, or reviewed by clinical experts, faculty, residents, or other domain specialists [30-33,35,37,38,40-45,49]. Human expertise informed scenario writing, checklist or rubric design, natural language processing rules, and other chatbot response logic. Only 1 (5%) study explicitly described cases as being developed by an LLM using contextual prompts [36]. Overall, case development remained largely a human-authored process, with AI more commonly used as an interaction and/or scoring mechanism rather than as a generator of clinical case content.

#### Platform, Access, and Frontend Interactions

Frontend design ranged from simple text-based chat interfaces to avatar-based immersive virtual environments. Fourteen (67%) of 21 studies used primarily text- or chat-based interfaces, making this the most common display format overall [31,35-41,44-47,49,50]. These systems presented the simulated patient through written dialogue and allowed learners to type questions in a conversational format. Text-based interfaces were especially common among recent LLM-enabled systems, suggesting that newer AI-SSPs prioritized conversational fluidity over visual embodiment.

Seven (33%) studies incorporated avatars, patient images, or other visual representations to add realism to the simulated encounter [30,32-34,42,43,48]. These systems were common before the wide adoption of LLMs and often included patient portraits, animated avatars, or graphical interfaces for selecting clinical actions beyond conversations. One (5%) study in particular used an even more immersive avatar-based virtual reality (VR) environment, in which learners wore vests and a VR headset to interact with the AI-SSP in a 3D simulated clinical setting [48]. Overall, earlier systems tended to be more visually elaborate but less conversationally flexible, whereas newer LLM-enabled systems were visually simpler but allowed more open-ended dialogue.

Most systems were delivered through online or computer-based platforms, although access details were inconsistently reported. Three systems were hosted offline, including earlier virtual patient systems [30,34,48]. Two studies used smartphone or tablet-based devices [33,44]. One study used a VR headset to support embodied interaction in an immersive environment [48]. The included studies suggest that AI-SSP frontend design has evolved from visually rich, platform-specific simulation environments toward more lightweight, chat-based interfaces that can be deployed through web, mobile, or existing LLM platforms.

### Clinical Competency Assessment

#### Clinical Skills Assessed

Table 3 summarizes the clinical competencies each included study assessed. Across the 21 studies, the most commonly assessed domain was history-taking or clinical inquiry, which was assessed in 11 (52%) studies. Diagnostic accuracy was assessed in 8 (38%) studies, and communication, empathy, professionalism, or counseling was also assessed in 8 (38%) studies. Only 4 (19%) studies assessed clinical examination or laboratory test selection, and only 2 (10%) evaluated disease management and treatment planning skills. Temporally, the focus of assessment also shifted from more structured clinical reasoning tasks, such as diagnosis and information gathering, toward more interactional competencies, including communication, empathy, counseling, and professionalism, largely mirroring the broader transition from rule-based virtual patient systems to more fluid, conversational LLM-enabled SSPs.

**Table 3.** AI-enabled simulated standardized patient competency assessment approaches.^a^

Study ID	Competency domains assessed	Quantitative scoring approach	Score derivation or metric	Score accuracy or reliability evaluation	Qualitative feedback
Courteille et al, 2008 [30]	History-taking; examination or laboratory selection; diagnosis	Checklist coverage+binary diagnosis	Percentage of required history questions or laboratories ordered; correct diagnosis; and completion time versus expert checklist.	Not formally assessed for automated scoring accuracy.	No automatic in-system feedback; and paper case summary provided post-session.
Oliven et al, 2011 [31]	Clinical skills or OSCE^b^; history, examination, and laboratories or imaging	Checklist coverage	Percentage of mandatory items identified or asked; item difficulty; discrimination; and Cronbach α computed.	Internal consistency: VP-OSCE^c^ α=0.82‐0.89 versus human OSCE α=0.65‐0.74; Pearson *r*=0.68‐0.71 with human OSCE.	Practice-mode gap feedback: asked versus missed or incorrect mandatory items; suppressed in examination mode.
Bond et al, 2019 [32]	High-value care; history, examination, diagnosis, testing, treatment	Weighted checklist+domain scores	NLP^d^ maps questions to prelabeled content; must ask=3 points, nice to ask=1 point; diagnosis, treatment, or testing scores.	Faculty versus platform rankings Spearman ρ mean=0.80; human-human ICC^e^ approximately 0.84‐0.85; direct human-machine IRR^f^ not computable.	Real-time pie chart plus end-of-case credit labels, content areas, transcript, and avatar-response appropriateness.
Maicher et al, 2019 [33]	Information gathering or history-taking	Checklist coverage	Essential history elements covered/21×100; subscale percentage for HOPI^g^, PMH^h^, FH^i^, and SH^j^.	ChatScript versus 3 faculty: no overall score difference (*P*=.07); all-rater agreement 82.6%; ChatScript categorization approximately 87%.	Automated session report: covered versus missed essential elements by history category plus expert model approach.
Yang et al, 2019 [34]	Medical knowledge; procedural communication; and investigation or diagnosis	Multicomponent competency scores	Right or wrong quiz; missed mandatory inquiry topics; and investigations or diagnoses compared with case possibilities.	Expert ground-truth overlap 75%‐88.9%; interactive accuracy 83.4%‐88.6% versus baseline 70.6%‐77.8%; CNN^k^ accuracy 0.85.	On-screen unasked-topic overlay; final student versus correct or possible investigation and diagnosis sets with detail view.
Lee et al, 2022 [35]	Physiotherapy clinical questioning or reasoning; empathy	Penalty or bonus score	Score out of 100; penalties for missed questions, red or yellow flags, consent, difficult questions; and empathy bonus.	No human agreement validation reported; intent mismatches manually corrected during testing.	Automated report after each attempt: missed or scored inputs with topic labels and example phrasings.
Brugge et al, 2024 [36]	Clinical decision-making or history-taking	Rubric or Likert scale	ChatGPT scores CRI-HTI^l^: 8 items across 3 domains, each 1‐5.	Human-human ICC=0.924 for transcript ratings; no direct AI-human agreement reported.	Feedback arm received 2-sentence justification per CRI-HTI item plus 3 personalized improvement suggestions.
Holderried et al, 2024 [37]	History-taking	Checklist coverage (completed by humans)	No in-tool quantitative competency score; manual post hoc QAP^m^ or script coverage analysis and CUQ^n^ usability score.	Manual analysis only; no kappa or ICC reported.	Unclear r or limited: UI^o^ showed feedback button, but feedback content or logic not described.
Holderried et al, 2024 [38]	History-taking completeness	Checklist or category coverage	GPT-4 returns yes or no JSON for 45 illness script categories; parsed to compute coverage %.	GPT-4 versus human rater Cohen κ=0.832 overall; several low-prevalence or overlapping categories <0.6.	Immediate GPT-4 structured narrative report: what was done well and missed items by history category.
Li et al, 2024 [39]	Clinical inquiry	Weighted checklist coverage	Aspect items (0.3)+specific information items (0.7); Llama-13B ensemble voting; normalized 0‐1.	Versus expert grading: Spearman *r*=0.655‐0.954; Pearson *r*=0.765‐0.943 across cases.	LLM^p^ writer generates examination report with explanatory advice from score results and conversation history.
Chiu et al, 2025 [40]	Breaking bad news; communication	Rubric score	ChatGPT scores SPIKES categories: 6 domains×5 points=30; with and without rubric.	ChatGPT overscored versus faculty (means 27.92/24.08 versus 16.79); significant differences; prompt or rubric dependent.	ChatGPT narrative feedback by SPIKES category: strengths, areas for improvement, and overall summary.
Gorcilov et al, 2025 [41]	Case evaluation and management plan	Numerical LLM score	LLM scored evaluation and plan per candidate; and rubric or maximum not reported.	Versus experts: Pearson r=0.88/0.76 for history, examination, or investigations; 0.69 and 0.71 for assessment and plan.	LLM-generated narrative feedback; and prompting and feedback logic not fully reported.
Haut et al, 2025 [42]	Serious illness communication; 3E^q^ skills	Classifier or process metrics+human outcome score	Real-time 3E skill labels and process metrics; RCT^r^ outcome separately human rated on 0‐1 scale.	Skill classifier accuracy=72%, *F*_1_=51%; and human outcome-rubric ICC=0.882.	Post-module GPT suggestions using transcript, 3E annotations, ACT^s^ context, and examples; and detailed PDF with excerpts and highlights.
Haut, 2025 [43]	Serious illness communication; 3E skills	Classifier or process metrics+human-rated rubric	Hybrid rule+ BERT^t^ 3E tags drive module progression; 18-item human-rated QSUM^u^ outcome normalized 0‐1.	BERT classifier accuracy=72%, *F*_1_=51%; human-human ICC=0.882 across 4 reviewers.	Feedback page: what done well, missed opportunities, actionable suggestions, color-coded transcript, and PDF report.
Liu et al, 2025 [44]	History-taking across multicase scenarios	Item-level checklist score	66/59/67 case items worth 0.5‐2 points; total 70; LLM scoring with decomposition and verification.	AI-human ICC=0.978/0.923/0.972 by case; item consistency 95.75%‐97.13%; cross-model consistency high.	Structured transparent report: dialogue, total score, category completeness, scored or missed items, citations, and rationale.
Qian et al, 2025 [45]	Case-based clinical reasoning	Key-point coverage	LLM given case scoring criteria; score=percentage of key points elicited; max 5‐8 key points per case.	Versus 4 experts: ICC=0.78‐0.82; Pearson *r*=0.67‐0.90; LLM initially about 12 pp higher, improved after 10 pp calibration.	No automatic qualitative feedback to learners reported.
Zheng et al, 2025 [46]	Clinical communication	Fuzzy categorical labels	Per-utterance labels for professionalism, medical relevance, ethical behavior, and contextual distraction with confidence.	Held-out test versus human majority vote: accuracy or weighted *F*_1_-score of approximately 0.82‐0.84; hybrid model outperformed baselines.	No learner-facing narrative feedback; outputs are fuzzy evaluation labels.
Zhou et al, 2025 [47]	Diagnostic accuracy in pulmonary disease	Diagnostic accuracy	Diagnostic accuracy reported post hoc as research outcome; not described as learner-facing automatic scoring.	Observational analysis only; no interrater or AI-human scoring agreement reported.	No automatic qualitative feedback reported; targeted feedback noted as future direction.
Chen, 2026 [48]	Empathic obstetric nursing communication	Rubric score	ECP-RS^v^: 5 empathy domains rated 0‐2; total /10 using NLP and speech or paraverbal analysis.	AI versus educator average ICC=0.81 single-measure, 0.89 average-measure; human ICCs 0.82‐0.88.	Personalized feedback by empathy domain: strengths, improvements, strategies for language, tone, pacing, and context.
Hicke et al, 2026 [49]	Clinical interviewing; history-taking; and communication	Hybrid rubric+checklist	MIRS^w^ items rated 1‐5 with anchored prompts; checklist completion present, absent, or unknown with excerpts.	External OSCE validation: exact accuracy 32.5%, off-by-one 64.1%, thresholded accuracy 87.0%.	Post-encounter itemized strengths or gaps with evidence quotes, suggestions, checklist feedback, and Learning Hub scaffolds.
Reynolds et al, 2026 [50]	Pharmacy counseling competencies	Rubric or ordinal scores	AI grading agent scores SME^x^-developed counseling rubric categories; categorical or ordinal scores.	AI versus human consensus Pearson *r* varied: −0.037 to 0.717; weaker for inference-heavy categories.	No automatic qualitative feedback described; rubric scores only.

a For each study, the table reports the competency domains assessed, quantitative scoring approach, score derivation or metric, evaluation of scoring accuracy or reliability, and whether qualitative feedback was provided.

b OSCE: objective structured clinical examination.

c VP-OSCE: virtual patient–objective structured clinical examination.

d NLP: natural language processing.

e ICC: intraclass correlation coefficient.

f IRR: interrater reliability.

g HOPI: history of present illness.

h PMH: past medical history.

i FH: family history.

j SH: social history.

k CNN: convolutional neural network.

l CRI-HTI: clinical reasoning indicators for history-taking.

m QAP: question-answer pair.

n CUQ: Chatbot Usability Questionnaire.

o UI: user interface.

p LLM: large language model.

q 3E: empathize, be explicit, and empower.

r RCT: randomized controlled trial.

s ACT: Advanced Communication Training.

t BERT: bidirectional encoder representations from transformers.

u QSUM: Quality of Communication questionnaire summary score.

v ECP-RS: Empathic Communication and Perspective-Taking Rating Scale.

w MIRS: Medical Interview Rating Scale.

x SME: subject matter expert.

#### Quantitative Scoring Approaches and Accuracy Validation

Although all included studies generated some form of learner performance measure, the source of scoring varied across studies. In 3 (14%) systems, the AI-SSP functioned primarily as the simulated patient, while performance was scored by human faculty, raters, or coders after the encounter using transcripts [36,42,43,48]. In other studies, scoring was automated by the AI-SSP system itself [38,39,44-46,49,50]. Several studies used both scoring approaches, and AI-generated scores were compared directly with human-led results [32,40,44,45,48-50].

Checklist-based scoring was the most common strategy. Ten (48%) studies used this approach where they translated expert-defined case content into required history-taking questions and clinical actions, then scored performance as item coverage, percent completion, or weighted checklist scores [30-33,37-39,44,45,49]. Nine (43%) studies used rubric-based or Likert scale scoring, particularly for communication-intensive competencies, such as empathy, professionalism, counseling, clarity, and ethical appropriateness [35,36,40,42,43,46,48-50]. A smaller number of studies used hybrid scoring formats, combining checklist completion with rubric ratings, classifier-generated process metrics, quizzes, diagnosis accuracy scores, or missed mandatory topic counts [34,42,43,49].

Where scores were AI generated, their accuracy and reliability were evaluated unevenly across the literature. Several studies did not formally validate automated scoring against a human benchmark [30,35,37,47], while others relied on internal consistency, classifier performance, or post hoc learning outcomes. Among studies comparing AI-generated scores with human ratings, the results were generally promising but variable. Correlation-based validation showed moderate to strong associations in Bond et al (Spearman ρ=0.80) [32], Li et al (Spearman *r*=0.66‐0.95 and Pearson *r*=0.77‐0.94) [39], Gorcilov et al (Pearson *r*=0.69‐0.88) [41], and Qian et al (Pearson *r*=0.67‐0.90) [45]. However, Reynolds et al [50] reported wider variability across checklist categories, with correlations ranging from negative values to 0.72.

In addition to correlation tests, agreement and reliability analyses similarly showed mixed but overall positive findings. Holderried et al [38] reported strong GPT-4 versus human agreement, with Cohen κ of 0.832 across 106 conversations and 45 categories [38]. Liu et al [44] reported high human-AI agreement across 3 cases, with intraclass correlation coefficients (ICCs) of 0.92 to 0.98 and item-level consistency of 95.75% to 97.13% [44]. Qian et al [45] reported ICCs of 0.78 to 0.82 after adjusting for consistent differences, while Chen [48] reported an AI versus educator average ICC of 0.81, comparable to human-human educator ICCs of 0.82 to 0.88. Classification-based evaluations were more variable: Haut [42,43] reported bidirectional encoder representations from transformers classifier accuracy of 72% and an *F*_1_-score of 51%, Zheng et al [46] reported accuracy of 0.82 to 0.84 and a weighted *F*_1_-score of 0.82 to 0.84, and Hicke et al [49] reported exact accuracy of 32.5%, off-by-one accuracy of 64.1%, and thresholded accuracy of 87.0%.

Several studies also identified important AI-based scoring limitations. Chiu et al [40] found that ChatGPT scores were substantially higher than faculty scores and sensitive to prompt design, while Qian et al [45] reported that LLMs were consistently around 12 percentage points higher than expert scores before calibration. Reynolds found that AI-human correlations varied widely by rubric category, including negative correlations for certain counseling-related items [50]. Although human-human reliability was sometimes reported as a benchmark [32,36,42,43,48], it was not consistently used to determine whether automated scoring achieved human-level performance [32,36,42,43,48]. Overall, while several systems demonstrated moderate to strong alignment with human ratings, validation approaches remained inconsistent, and evidence for reliably automated scoring was still limited.

#### Qualitative Feedback Approaches

Qualitative feedback was common across studies but varied substantially in specificity and actionability. Earlier systems often provided checklist- or gap-based feedback, identifying missed questions, omitted clinical actions, or incomplete case elements [31-35,40,49]. Newer LLM-enabled systems increasingly generated narrative feedback that included strengths, weaknesses, transcript-based examples, and personalized improvement suggestions [36,38,40-43,48]. Several studies used hybrid feedback approaches that combined checklist completion and narrative comments [39,42,43,48,49]. Overall, feedback evolved from relatively simple “what was missed” summaries toward more individualized coaching focused on communication, clinical reasoning, and next steps for improvement.

### AI-SSP Product Evaluation and Evidence Maturity

Beyond scoring accuracy, studies also evaluated AI-SSP systems using a range of product-level outcomes, including learner experience, AI-SSP response quality, and learning outcomes. Several studies reported high learner satisfaction, perceived usefulness, ease of navigation, or willingness to recommend the system [30,32-34,37,38,40,42-44,49]. Response quality and realism were also commonly evaluated, assessing whether AI-SSP responses were plausible, realistic, or aligned with expected case content [30,32,38,39,41-43]. Evidence for downstream skills improvement was more limited, with only 6 (6/21, 29%) studies assessing this outcome [36,40,42,43,48,49].

**Figure 3.**
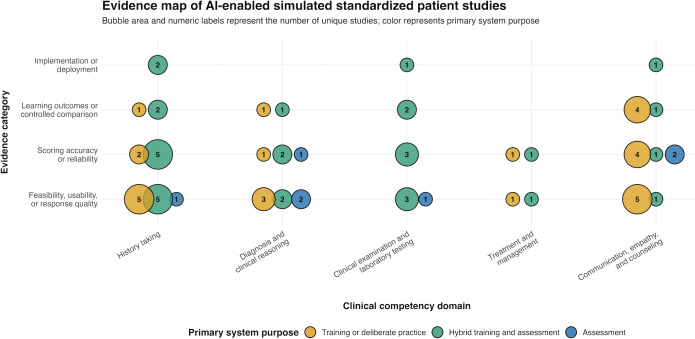
Evidence gap map of AI-enabled simulated standardized patient studies. The figure maps the distribution of included studies across 5 clinical competency domains—history-taking; diagnosis and clinical reasoning; clinical examination and laboratory testing; treatment and management; and communication, empathy, and counseling—and 4 categories of evidence maturity: feasibility, usability, or response quality; scoring accuracy or reliability; learning outcomes or controlled comparison; and implementation or deployment. Each bubble represents the number of unique studies at a given intersection of competency domain, evidence category, and primary system purpose. Bubble area is proportional to the number of studies, and the numeric label inside each bubble gives the exact count. Bubble color indicates the primary purpose of the system: training or deliberate practice, hybrid training and assessment, or assessment. The unit represented is the study rather than the unique AI-SSP system; therefore, separate publications evaluating the same system are counted separately. Studies could also contribute to more than one competency domain and more than one evidence category. Accordingly, counts are not mutually exclusive and should not be summed across cells.

As illustrated in Figure 3, the available evidence was concentrated primarily in early-stage product evaluation rather than learning outcomes, controlled comparisons, or sustained implementation. Feasibility, usability, or response quality was the most frequently assessed evidence category, reported in 18 (86%) studies, followed closely by scoring accuracy or reliability in 16 (76%) studies. In contrast, only 8 (38%) studies evaluated learning outcomes or incorporated a controlled comparison, and this evidence was unevenly distributed across competency domains, with the strongest concentration in communication-related skills and history-taking. Evidence was particularly limited for treatment and management and for clinical examination or laboratory testing, suggesting that the literature has not yet established whether AI-SSPs can support assessment across the full clinical workflow. Only 2 (10%) studies reported evidence of implementation or deployment, indicating that most systems remain at the pilot, proof-of-concept, or validation stage. Studies of training-oriented systems contributed mainly to feasibility, usability, and early learning evidence, whereas studies of hybrid training and assessment systems demonstrated the broadest evidence profile, accounting for all identified implementation evidence and a substantial share of scoring validation evidence. Assessment-focused systems were more narrowly concentrated in scoring accuracy and reliability, with comparatively little evidence on learning effects or real-world adoption. Overall, the evidence gap map shows that the field has made meaningful progress in demonstrating technical feasibility and initial scoring accuracy and reliability, but stronger evidence is still needed on educational effectiveness, sustained use, scalability, integration into high-stakes assessment, and performance across the full range of clinical competency domains.

## Discussion

This scoping review aimed to map how AI-enabled SSP systems have been used for clinical competency assessment, with attention to system architecture, interaction design, assessed clinical competencies, scoring and feedback approaches, and evidence maturity. Across 21 studies and 20 unique AI-SSP systems, we found that the field is expanding rapidly, with 15 (71%) studies published in 2024 or later and a clear temporal shift from earlier rule-based virtual patient systems to LLM-enabled conversational platforms after 2022. The evidence base remained concentrated in high-income academic settings, focused primarily on medical students, and dominated by pilot, feasibility, and proof-of-concept studies rather than large-scale implementation research. Although all included studies produced some form of competency-related performance measure, AI-SSPs were most often positioned as formative learning tools rather than formal assessment systems, with 9 systems focused primarily on deliberate practice, 6 combining training and assessment, and 5 explicitly assessment-oriented. Across studies, history-taking was the most commonly assessed competency, while laboratory tests, diagnosis, treatment planning, communication, empathy, and professionalism were assessed less consistently. Quantitative scoring and qualitative feedback were common but heterogeneous: checklist-based scoring was most frequent, rubric-based scoring was used mainly for communication-intensive competencies, and validation of automated scoring remained inconsistent. Overall, our findings suggest that AI-SSPs are increasingly capable of supporting scalable and repeatable competency assessment, but the current literature is more mature in demonstrating feasibility and usability than in establishing validity and implementation readiness.

Previous research on virtual patients has shown that digital patient simulations can give learners consistent clinical cases to practice with, helping them build medical knowledge, clinical reasoning skills, and confidence [15,16]. However, these systems have historically functioned primarily as educational simulations rather than scalable competency assessment infrastructures. Similarly, recent reviews of LLM-based virtual patients describe a newer wave of systems focused on conversational realism, history-taking, and medical training, reflecting broader enthusiasm for generative AI in health professions education [17-19]. Our findings build on this literature by showing that AI-SSPs are beginning to move beyond simulation alone toward competency measurement but remain in a transitional stage. The shift from rule-based virtual patient systems to LLM-enabled platforms has improved conversational fluency, responsiveness, and perceived realism, but most systems still rely on human-authored cases, structured prompts, and rubrics, which represent important guardrails against hallucinations. Current AI-SSPs are not fully agentic assessors of clinical competence, but semistructured assessment environments in which generative AI is layered onto human-defined clinical content and scoring logic.

The concentration of assessed competencies also has important implications for how AI-SSPs should be interpreted as assessment tools. Consistent with the broader virtual patient literature, the included systems most often assessed history-taking, diagnosis, and communication-related skills, domains that map naturally onto text-based or conversational interaction [16,17,51]. However, fewer studies assessed physical examination, laboratory or investigation selection, and management planning. This pattern suggests that current AI-SSPs are strongest for evaluating how learners gather information and communicate with patients but less mature for assessing the full clinical workflow from examination to diagnostic interpretation and treatment decision-making. For formative education, this may be sufficient and even valuable. For competency assessment, however, the narrow domain coverage raises concerns about how a learner may perform well in an AI-SSP conversation without demonstrating the broader set of skills required for safe and effective clinical care.

In terms of automated scoring of performances, our review showed that checklist-based scoring was most common, reflecting the need to convert complex encounters into observable items. Rubric-based scoring was used mainly for communication-intensive domains, where performance depended not only on whether an item was mentioned but also on how the learner expressed empathy, professionalism, clarity, or ethical reasoning. Several included studies reported moderate to strong agreement between AI-generated and human or expert scores, suggesting that automated scoring may reduce faculty burden and enable more frequent formative assessment. However, there is also evidence of consistent score inflation, prompt sensitivity, and variable performance across rubric categories in other studies. This pattern mirrors findings from the comparison of AI and human evaluation of the same video-recorded OSCE performances by Tekin et al [52], in which AI models consistently assigned higher scores than human evaluators across clinical skills, including a mean score of 28.23 versus 25.25 for intramuscular injection. Similar tendencies appeared in our included studies: Chiu et al [40] found that ChatGPT scores were substantially higher than faculty scores, and Qian et al [45] reported that LLM scores were approximately 12 percentage points higher than expert scores before calibration. These findings suggest that AI scoring may be scalable and internally consistent, but not necessarily equivalent to human judgment. Additionally, while several studies reported high correlations or ICCs between AI and human raters, evidence regarding construct validity, fairness, and suitability for high-stakes summative competency assessment remains limited. Stronger validation should therefore include transparent scoring logic, calibration against expert benchmarks, comparison with human-human reliability, and domain-specific evaluation rather than reliance on aggregate correlations alone.

The overall evidence maturity of AI-SSPs remains limited compared to their technical promise. This pattern is consistent with broader reviews of digital simulation and AI in health professions education, which have found that usability, satisfaction, and short-term learning outcomes are more commonly evaluated than large-scale trials, implementation, or real-world clinical impact [22]. In this review, most studies demonstrated that AI-SSPs were feasible, acceptable, realistic, or capable of producing structured scores and feedback, but fewer examined whether they improved clinical competency, could be integrated into existing curricula, or performed reliably across various types of users, especially in low-resource contexts. As promising prototypes do not automatically translate into scalable assessment infrastructure, future studies should move beyond product-level feasibility toward larger-scale validation, assess longitudinal learning outcomes, and test in diverse geographic, socioeconomic, and health professional contexts.

A further implementation concern is the need for AI safety protections when AI-SSPs are used for assessment, especially for future high-stakes summative assessments beyond formative ones. As these systems may collect detailed learner transcripts, performance scores, and interaction logs, institutions will need clear data governance policies covering consent, storage, and secondary use of assessment data [53]. Technical guardrails are also needed to prevent unsafe or inappropriate patient responses, protect confidential case content, and ensure that automated feedback does not provide misleading clinical guidance [54]. These governance and safety issues are central to whether AI-SSPs can be trusted as assessment tools. Furthermore, assessment-oriented AI-SSPs also create risks related to new ways of cheating, plagiarism, and the authenticity of performance, particularly if learners can use external AI tools, reuse shared prompts, or access case materials outside the assessment setting. Future systems should therefore include procedural safeguards such as secure testing environments, controlled access to cases and rubrics, and identity verification when appropriate.

This review has several limitations. First, it synthesizes heterogeneous study designs and tool implementations, many of which were small-scale pilots or descriptive prototypes rather than fully validated systems. Second, the rapid evolution of LLM technologies also means that some findings may become outdated as models and deployment practices change. Third, most included studies originated in high-income settings, limiting generalizability to low- and middle-income health systems where SSPs may offer the greatest potential value. At the same time, this review has important strengths. It is, to our knowledge, the first to systematically characterize AI-led SSPs for clinical competency “assessment.” We explored frontend design, backend architecture, and evaluation methods, offering a holistic view of how these tools are currently constructed and assessed. By identifying common architectural patterns and persistent gaps, this review provides a conceptual framework for future SSP development and highlights key design and validation priorities to advance scalable, reliable clinical competency assessment.

In conclusion, AI-enabled SSPs represent an important emerging direction for clinical competency assessment, but the field remains in an early stage of development. This review shows that recent advances in LLMs have accelerated a shift from rule-based virtual patient systems toward more conversational and flexible assessment environments. At the same time, most systems continue to rely on human-authored cases, structured prompts, checklists, rubrics, and other guardrails, underscoring that AI-SSPs are not autonomous assessors of clinical competence. The current evidence base suggests strongest readiness for formative assessment of history-taking, communication, and selected clinical reasoning skills, while broader clinical workflows, including clinical examination, laboratory tests, treatment planning, and management, remain less consistently evaluated. For AI-SSPs to move from promising prototypes to trustworthy assessment infrastructure, future studies must establish stronger evidence of reliability, validity, fairness, and implementation feasibility across diverse learners, professions, and health system contexts. With rigorous validation and responsible governance, AI-SSPs could help expand access to more frequent, standardized, and scalable competency assessments, particularly in settings where traditional SP-based assessments are difficult to sustain.

## Supplementary material

10.2196/92826 Multimedia Appendix 1 Complete database search strategies. Detailed search strategies, including databases and platforms searched, search dates, keywords, controlled vocabulary terms, Boolean operators, and limits applied.

10.2196/92826 Checklist 1 PRISMA-S checklist.

## Abbreviations

AI: artificial intelligence
GAIDeT: Generative AI Delegation Taxonomy
Gen-AI: generative artificial intelligence
ICC: intraclass correlation coefficient
LLM: large language model
OSCE: objective structured clinical examination
PRISMA: Preferred Reporting Items for Systematic Reviews and Meta-Analyses
PRISMA-S: Preferred Reporting Items for Systematic Reviews and Meta-Analyses Literature Search Extension
PRISMA-ScR: Preferred Reporting Items for Systematic Reviews and Meta-Analyses Extension for Scoping Reviews
SP: standardized patient
SSP: simulated standardized patient
UHC: Universal Health Coverage
VR: virtual reality
