# Supplementary material for: AI for Clinical Competency Assessment: Scoping Review of Methods and Applications
Source: JMIR Med Educ. 2026 Aug 14;12:e92826. doi: 10.2196/92826 (PMC13478279; doi:10.2196/92826)
Supplement: Supplementary file 2 [file mededu-v12-e92826-s001.docx]

**Multimedia Appendix Supplementary File:** **Scoping Review Search Terms and Records**

MEDLINE (National Library of Medicine, OVID)

1261 records - May 7, 2026

Ovid MEDLINE(R) and Epub Ahead of Print, In-Process, In-Data-Review & Other Non-Indexed Citations and Daily 1946 to May 06, 2026

1

"Patient Simulation"/ OR (vignett* OR clinical scenario? OR clinical case? OR case scenario? OR simulat* case? OR "case simulation?" OR ("case based" adj (scenario? OR learning OR education* OR instruction* OR teaching)) OR (simulat* adj4 (patient? OR clinical)) OR (virtual adj4 patient?) OR patient avatar? OR standardized patient? OR standardised patient?).ti,ab,kf,kw

2

exp "Artificial Intelligence"/ OR (artificial intelligence OR ai OR genAI OR support vector? OR large language OR language model* OR llm OR llms OR chatbot? OR chatgpt OR gpt OR claude OR transformer? OR conversational agent? OR virtual agent? OR virtual assistant? OR intelligent agent? OR natural language process* OR expert system? OR machine learning OR foundation model* OR dialog* system?).ti,ab,kf,kw

3.

"Educational Measurement"/ OR "Clinical Competence"/ OR "Professional Competence"/ OR "Competency-Based Education"/ OR "Education, Medical"/ OR "Education, Medical, Undergraduate"/ OR exp "Education, Nursing"/ OR "Clinical Reasoning"/ OR (osce? OR objective structured OR (student? adj4 (assess* OR evaluat*)) OR ((medical OR nursing OR clinical OR biomedical) adj (education OR training OR curricul*)) OR feedback OR competenc* OR skill? OR clinical reasoning OR clinical judgement OR clinical proficiency OR clinical knowledge OR diagnostic accuracy OR diagnostic reasoning).ti,ab,kf,kw

(1 AND 2 AND 3)

Embase (Elsevier, embase.com)

1363 records - May 7, 2026

Advanced Search

Remove Embase mapping options

Sources: Embase (1974 to present)

#1

'patient simulation'/exp OR 'virtual patient'/de OR 'vignette'/de OR 'case based learning'/de OR (vignett* OR "clinical scenario$" OR "clinical case$" OR "case scenario$" OR "simulat* case$" OR "case simulation$" OR ("case based" NEXT/1 (scenario$ OR learning OR education* OR instruction* OR teaching)) OR (simulat* NEAR/4 (patient$ OR clinical)) OR (virtual NEAR/4 patient$) OR "patient avatar$" OR "standardized patient$" OR "standardised patient$"):ab,ti,kw

#2

'artificial intelligence'/exp OR 'chatbot'/exp OR 'virtual assistant'/de OR 'machine learning'/exp OR 'large language model'/de OR 'natural language processing'/de OR 'expert system'/de OR ("artificial intelligence" OR AI OR genAI OR "support vector$" OR "large language" OR "language model*" OR llm OR llms OR chatbot$ OR chatgpt OR claude OR gpt OR transformer$ OR "conversational agent$" OR "virtual agent$" OR "virtual assistant$" OR "intelligent agent$" OR "natural language process*" OR "expert system$" OR "machine learning" OR "foundation model*" OR "dialog* system$"):ab,ti,kw

#3

'clinical competence'/de OR 'nursing competence'/de OR 'professional competence'/de OR 'competency-based education'/exp OR 'medical education'/de OR 'clinical education'/de OR 'nursing education'/de OR 'objective structured clinical examination'/de OR 'clinical skill'/de OR 'clinical reasoning'/exp OR 'diagnostic accuracy'/de OR (osce$ OR "objective structured" OR (student$ NEAR/4 (assess* OR evaluat*)) OR ((medical OR nursing OR clinical OR biomedical) NEXT/1 (education OR training OR curricul*)) OR feedback OR competenc* OR skill$ OR "clinical reasoning" OR "clinical judgement" OR "clinical proficiency" OR "clinical knowledge" OR "diagnostic accuracy" OR "diagnostic reasoning"):ab,ti,kw

(#1 AND #2 AND #3)

Cumulative Index to Nursing and Allied Health Literature (CINAHL Complete, EBSCOhost)

356 records – May 7, 2026

Advanced search

S1

MH "Vignettes" OR MH "Patient Simulation" OR XB (vignett* OR "clinical scenario#" OR "clinical case#" OR "case scenario#" OR "simulat* case#" OR "case simulation#" OR ("case based" W0 (scenario# OR "learning" OR education* OR instruction* OR "teaching")) OR (simulat* N3 (patient# OR "clinical")) OR ("virtual" N3 patient#) OR "patient avatar#" OR "standardized patient#" OR "standardised patient#")

S2

MH "Artificial Intelligence+" OR XB ("artificial intelligence" OR "AI" OR "genAI" OR "support vector#" OR "large language" OR "language model*" OR "llm" OR "llms" OR chatbot# OR "chatgpt" OR "claude" OR "gpt" OR transformer# OR "conversational agent# " OR "virtual agent#" OR "virtual assistant#" OR "intelligent agent#" OR "natural language process*" OR "expert system#" OR "machine learning" OR "foundation model*" OR "dialog* system#")

S3

MH "Educational Measurement" OR MH "Clinical Competence+" OR MH "Professional Competence"

OR MH "Education, Competency-Based" OR MH "Education, Clinical" OR MH "Education, Health Sciences+" OR MH "Clinical Reasoning" OR MH "Diagnostic Reasoning" OR XB (osce# OR "objective structured" OR (student# N3 (assess* OR evaluat*)) OR ((medical OR nursing OR clinical OR biomedical) W0 ("education" OR "training" OR curricul*)) OR "feedback" OR competenc* OR skill# OR "clinical reasoning" OR "clinical judgement" OR "clinical proficiency" OR "clinical knowledge" OR "diagnostic accuracy" OR "diagnostic reasoning")

(S1 AND S2 AND S3)

Education Source (EBSCOhost)

187 records – May 5, 2026

Advanced Search

S1

DE "Vignettes (Teaching technique)" OR DE "Simulation methods in medical education" OR DE "Simulated patients" OR DE "Case method (Teaching)" OR TI (vignett* OR "clinical scenario#" OR "clinical case#" OR "case scenario#" OR "simulat* case#" OR "case simulation#" OR ("case based" W0 (scenario# OR "learning" OR education* OR instruction* OR "teaching")) OR (simulat* N3 (patient# OR "clinical")) OR ("virtual" N3 patient#) OR "patient avatar#" OR "standardized patient#" OR "standardised patient#") OR AB (vignett* OR "clinical scenario#" OR "clinical case#" OR "case scenario#" OR "simulat* case#" OR "case simulation#" OR ("case based" W0 (scenario# OR "learning" OR education* OR instruction* OR "teaching")) OR (simulat* N3 (patient# OR "clinical")) OR ("virtual" N3 patient#) OR "patient avatar#" OR "standardized patient#" OR "standardised patient#") OR KW (vignett* OR "clinical scenario#" OR "clinical case#" OR "case scenario#" OR "simulat* case#" OR "case simulation#" OR ("case based" W0 (scenario# OR "learning" OR education* OR instruction* OR "teaching")) OR (simulat* N3 (patient# OR "clinical")) OR ("virtual" N3 patient#) OR "patient avatar#" OR "standardized patient#" OR "standardised patient#")

S2

DE "Artificial intelligence" OR DE "Generative artificial intelligence" OR DE "Generative pre-trained transformers" OR DE "ChatGPT" OR DE "Chatbots" OR DE "Virtual agents" OR DE "Intelligent agents" OR DE "Language models" OR DE "Natural language processing" OR DE "Machine learning" OR DE "Expert systems OR TI ("artificial intelligence" OR "AI" OR "genAI" OR "support vector#" OR "large language" OR "language model*" OR "llm" OR "llms" OR chatbot# OR "chatgpt" OR "claude" OR "gpt" OR transformer# OR "conversational agent# " OR "virtual agent#" OR "virtual assistant#" OR "intelligent agent#" OR "natural language process*" OR "expert system#" OR "machine learning" OR "foundation model*" OR "dialog* system#") OR AB ("artificial intelligence" OR "AI" OR "genAI" OR "support vector#" OR "large language" OR "language model*" OR "llm" OR "llms" OR chatbot# OR "chatgpt" OR "claude" OR "gpt" OR transformer# OR "conversational agent# " OR "virtual agent#" OR "virtual assistant#" OR "intelligent agent#" OR "natural language process*" OR "expert system#" OR "machine learning" OR "foundation model*" OR "dialog* system#") OR KW ("artificial intelligence" OR "AI" OR "genAI" OR "support vector#" OR "large language" OR "language model*" OR "llm" OR "llms" OR chatbot# OR "chatgpt" OR "claude" OR "gpt" OR transformer# OR "conversational agent# " OR "virtual agent#" OR "virtual assistant#" OR "intelligent agent#" OR "natural language process*" OR "expert system#" OR "machine learning" OR "foundation model*" OR "dialog* system#")

S3

DE "Educational tests & measurements" OR DE "Clinical competence" OR DE "Clinical education" OR DE "Medical education" OR DE "Allied health education" OR DE "Clinical medical education" OR DE "Nursing education" OR TI (osce# OR "objective structured" OR (student# N3 (assess* OR evaluat*)) OR ((medical OR nursing OR clinical OR biomedical) W0 ("education" OR "training" OR curricul*)) OR "feedback" OR competenc* OR skill# OR "clinical reasoning" OR "clinical judgement" OR "clinical proficiency" OR "clinical knowledge" OR "diagnostic accuracy" OR "diagnostic reasoning") OR AB (osce# OR "objective structured" OR (student# N3 (assess* OR evaluat*)) OR ((medical OR nursing OR clinical OR biomedical) W0 ("education" OR "training" OR curricul*)) OR "feedback" OR competenc* OR skill# OR "clinical reasoning" OR "clinical judgement" OR "clinical proficiency" OR "clinical knowledge" OR "diagnostic accuracy" OR "diagnostic reasoning") OR KW (osce# OR "objective structured" OR (student# N3 (assess* OR evaluat*)) OR ((medical OR nursing OR clinical OR biomedical) W0 ("education" OR "training" OR curricul*)) OR "feedback" OR competenc* OR skill# OR "clinical reasoning" OR "clinical judgement" OR "clinical proficiency" OR "clinical knowledge" OR "diagnostic accuracy" OR "diagnostic reasoning")

(S1 AND S2 AND S3)

ERIC (Educational Resource Information Center, EBSCOhost)

47 records – May 5, 2026

Advanced Search

S1

DE "Vignettes" OR ((DE "Simulation" OR DE "Computer Simulation") AND DE "Patients")

OR DE "Case Method (Teaching Technique)" OR TI (vignett* OR "clinical scenario#" OR "clinical case#" OR "case scenario#" OR "simulat* case#" OR "case simulation#" OR ("case based" W0 (scenario# OR "learning" OR education* OR instruction* OR "teaching")) OR (simulat* N3 (patient# OR "clinical")) OR ("virtual" N3 patient#) OR "patient avatar#" OR "standardized patient#" OR "standardised patient#") OR AB (vignett* OR "clinical scenario#" OR "clinical case#" OR "case scenario#" OR "simulat* case#" OR "case simulation#" OR ("case based" W0 (scenario# OR "learning" OR education* OR instruction* OR "teaching")) OR (simulat* N3 (patient# OR "clinical")) OR ("virtual" N3 patient#) OR "patient avatar#" OR "standardized patient#" OR "standardised patient#")

S2

DE "Artificial Intelligence" OR DE "Natural Language Processing" OR TI ("artificial intelligence" OR "AI" OR "genAI" OR "support vector#" OR "large language" OR "language model*" OR "llm" OR "llms" OR chatbot# OR "chatgpt" OR "claude" OR "gpt" OR transformer# OR "conversational agent# " OR "virtual agent#" OR "virtual assistant#" OR "intelligent agent#" OR "natural language process*" OR "expert system#" OR "machine learning" OR "foundation model*" OR "dialog* system#") OR AB ("artificial intelligence" OR "AI" OR "genAI" OR "support vector#" OR "large language" OR "language model*" OR "llm" OR "llms" OR chatbot# OR "chatgpt" OR "claude" OR "gpt" OR transformer# OR "conversational agent# " OR "virtual agent#" OR "virtual assistant#" OR "intelligent agent#" OR "natural language process*" OR "expert system#" OR "machine learning" OR "foundation model*" OR "dialog* system#")

S3

DE "Medical Education" OR DE "Nursing Education" OR DE "Allied Health Occupations Education" OR DE "Clinical Diagnosis" OR TI (osce# OR "objective structured" OR (student# N3 (assess* OR evaluat*)) OR ((medical OR nursing OR clinical OR biomedical) W0 ("education" OR "training" OR curricul*)) OR "feedback" OR competenc* OR skill# OR "clinical reasoning" OR "clinical judgement" OR "clinical proficiency" OR "clinical knowledge" OR "diagnostic accuracy" OR "diagnostic reasoning") OR AB (osce# OR "objective structured" OR (student# N3 (assess* OR evaluat*)) OR ((medical OR nursing OR clinical OR biomedical) W0 ("education" OR "training" OR curricul*)) OR "feedback" OR competenc* OR skill# OR "clinical reasoning" OR "clinical judgement" OR "clinical proficiency" OR "clinical knowledge" OR "diagnostic accuracy" OR "diagnostic reasoning")

(S1 AND S2 AND S3)

Inspec (Elsevier Engineering Information, EBSCOhost)

1087 records - May 7, 2026

Advanced Search

S1

(DE "computer simulation" OR DE "computer aided instruction" OR DE "computer based training") AND (DE "medical computing" OR DE "medical expert systems") OR TI (vignett* OR "clinical scenario#" OR "clinical case#" OR "case scenario#" OR "simulat* case#" OR "case simulation#" OR ("case based" W0 (scenario# OR "learning" OR education* OR instruction* OR "teaching")) OR (simulat* N3 (patient# OR "clinical")) OR ("virtual" N3 patient#) OR "patient avatar#" OR "standardized patient#" OR "standardised patient#") OR AB (vignett* OR "clinical scenario#" OR "clinical case#" OR "case scenario#" OR "simulat* case#" OR "case simulation#" OR ("case based" W0 (scenario# OR "learning" OR education* OR instruction* OR "teaching")) OR (simulat* N3 (patient# OR "clinical")) OR ("virtual" N3 patient#) OR "patient avatar#" OR "standardized patient#" OR "standardised patient#")

S2

DE "artificial intelligence" OR DE "chatbots" OR DE "generative AI" OR DE "generative adversarial networks" OR DE "large language models" OR DE "support vector machines" OR DE "machine learning" OR DE "transformers (machine learning)" OR DE "deep learning (artificial intelligence)" OR DE "multi-agent systems" OR DE "expert systems" OR TI ("artificial intelligence" OR "AI" OR "genAI" OR "support vector#" OR "large language" OR "language model*" OR "llm" OR "llms" OR chatbot# OR "chatgpt" OR "claude" OR "gpt" OR transformer# OR "conversational agent# " OR "virtual agent#" OR "virtual assistant#" OR "intelligent agent#" OR "natural language process*" OR "expert system#" OR "machine learning" OR "foundation model*" OR "dialog* system#") OR AB ("artificial intelligence" OR "AI" OR "genAI" OR "support vector#" OR "large language" OR "language model*" OR "llm" OR "llms" OR chatbot# OR "chatgpt" OR "claude" OR "gpt" OR transformer# OR "conversational agent# " OR "virtual agent#" OR "virtual assistant#" OR "intelligent agent#" OR "natural language process*" OR "expert system#" OR "machine learning" OR "foundation model*" OR "dialog* system#")

S3

DE "biomedical education" OR TI (osce# OR "objective structured" OR (student# N3 (assess* OR evaluat*)) OR ((medical OR nursing OR clinical OR biomedical) W0 ("education" OR "training" OR curricul*)) OR "feedback" OR competenc* OR skill# OR "clinical reasoning" OR "clinical judgement" OR "clinical proficiency" OR "clinical knowledge" OR "diagnostic accuracy" OR "diagnostic reasoning") OR AB (osce# OR "objective structured" OR (student# N3 (assess* OR evaluat*)) OR ((medical OR nursing OR clinical OR biomedical) W0 ("education" OR "training" OR curricul*)) OR "feedback" OR competenc* OR skill# OR "clinical reasoning" OR "clinical judgement" OR "clinical proficiency" OR "clinical knowledge" OR "diagnostic accuracy" OR "diagnostic reasoning")

(S1 AND S2 AND S3)

Web of Science Core Collection (Clarivate)

1,532 records May 7, 2026

All Editions

Advanced Search

More options – Exact search

#1

TI=(vignett* OR "clinical scenario$" OR "clinical case$" OR "case scenario$" OR "simulat* case$" OR "case simulation$" OR ("case based" NEAR/0 (scenario$ OR "learning" OR education* OR instruction* OR "teaching")) OR (simulat* NEAR/3 (patient$ OR "clinical")) OR ("virtual" NEAR/3 patient$) OR "patient avatar$" OR "standardized patient$" OR "standardised patient$")

#2

AB=(vignett* OR "clinical scenario$" OR "clinical case$" OR "case scenario$" OR "simulat* case$" OR "case simulation$" OR ("case based" NEAR/0 (scenario$ OR "learning" OR education* OR instruction* OR "teaching")) OR (simulat* NEAR/3 (patient$ OR "clinical")) OR ("virtual" NEAR/3 patient$) OR "patient avatar$" OR "standardized patient$" OR "standardised patient$")

#3

AK TI=(vignett* OR "clinical scenario$" OR "clinical case$" OR "case scenario$" OR "simulat* case$" OR "case simulation$" OR ("case based" NEAR/0 (scenario$ OR "learning" OR education* OR instruction* OR "teaching")) OR (simulat* NEAR/3 (patient$ OR "clinical")) OR ("virtual" NEAR/3 patient$) OR "patient avatar$" OR "standardized patient$" OR "standardised patient$")

#4

TI=("artificial intelligence" OR "AI" OR "genAI" OR "support vector$" OR "large language" OR "language model*" OR "llm" OR "llms" OR chatbot$ OR "chatgpt" OR "claude" OR "gpt" OR transformer$ OR "conversational agent$ " OR "virtual agent$" OR "virtual assistant$" OR "intelligent agent$" OR "natural language process*" OR "expert system$" OR "machine learning" OR "foundation model*" OR "dialog* system$")

#5

AB=("artificial intelligence" OR "AI" OR "genAI" OR "support vector$" OR "large language" OR "language model*" OR "llm" OR "llms" OR chatbot$ OR "chatgpt" OR "claude" OR "gpt" OR transformer$ OR "conversational agent$ " OR "virtual agent$" OR "virtual assistant$" OR "intelligent agent$" OR "natural language process*" OR "expert system$" OR "machine learning" OR "foundation model*" OR "dialog* system$")

#6

AK=("artificial intelligence" OR "AI" OR "genAI" OR "support vector$" OR "large language" OR "language model*" OR "llm" OR "llms" OR chatbot$ OR "chatgpt" OR "claude" OR "gpt" OR transformer$ OR "conversational agent$ " OR "virtual agent$" OR "virtual assistant$" OR "intelligent agent$" OR "natural language process*" OR "expert system$" OR "machine learning" OR "foundation model*" OR "dialog* system$")

#7

TI=(osce$ OR "objective structured" OR (student$ NEAR/3 (assess* OR evaluat*)) OR ((medical OR nursing OR clinical OR biomedical) NEAR/0 ("education" OR "training" OR curricul*)) OR "feedback" OR competenc* OR skill$ OR "clinical reasoning" OR "clinical judgement" OR "clinical proficiency" OR "clinical knowledge" OR "diagnostic accuracy" OR "diagnostic reasoning")

#8

AB=(osce$ OR "objective structured" OR (student$ NEAR/3 (assess* OR evaluat*)) OR ((medical OR nursing OR clinical OR biomedical) NEAR/0 ("education" OR "training" OR curricul*)) OR "feedback" OR competenc* OR skill$ OR "clinical reasoning" OR "clinical judgement" OR "clinical proficiency" OR "clinical knowledge" OR "diagnostic accuracy" OR "diagnostic reasoning")

#9

AK=(osce$ OR "objective structured" OR (student$ NEAR/3 (assess* OR evaluat*)) OR ((medical OR nursing OR clinical OR biomedical) NEAR/0 ("education" OR "training" OR curricul*)) OR "feedback" OR competenc* OR skill$ OR "clinical reasoning" OR "clinical judgement" OR "clinical proficiency" OR "clinical knowledge" OR "diagnostic accuracy" OR "diagnostic reasoning")

(#1 OR #2 OR #3) AND (#4 OR #5 OR #6) AND (#7 OR #8 OR #9)

Cochrane Central Register of Controlled Trials (Cochrane Library, Wiley)

154 records - May 7, 2026

Advanced Search

Search limits: turn off "Search word variations"

Search manager:

#1

(vignett* OR (clinical NEXT scenario?) OR (clinical NEXT case?) OR (case NEXT scenario?) OR (simulat* NEXT case?) OR (case NEXT simulation?) OR ("case based" NEXT (scenario? OR "learning" OR education* OR instruction* OR "teaching")) OR (simulat* NEAR/3 (patient? OR "clinical")) OR ("virtual" NEAR/3 patient?) OR (patient NEXT avatar?) OR (standardized NEXT patient?) OR (standardised NEXT patient?)):ti,ab,kw

#2

("artificial intelligence" OR "AI" OR "genAI" OR (support NEXT vector?) OR "large language" OR (language NEXT model*) OR "llm" OR "llms" OR chatbot? OR "chatgpt" OR "claude" OR "gpt" OR transformer? OR (conversational NEXT agent?) OR (virtual NEXT agent?) OR (virtual NEXT assistant?) OR (intelligent NEXT agent) OR ("natural language" NEXT process*) OR (expert NEXT system?) OR "machine learning" OR (foundation NEXT model*) OR (dialog* NEXT system?)):ti,ab,kw

#3

(osce? OR "objective structured" OR (student? NEAR/3 (assess* OR evaluat*)) OR ((medical OR nursing OR clinical OR biomedical) NEXT ("education" OR "training" OR curricul*)) OR "feedback" OR competenc* OR skill? OR "clinical reasoning" OR "clinical judgement" OR "clinical proficiency" OR "clinical knowledge" OR "diagnostic accuracy" OR "diagnostic reasoning"):ti,ab,kw

#1 AND #2 AND #3
